# Beyond Location Probability: Neural Correlates of Feature–Spatial Mapping Learning in Visual Search

**DOI:** 10.3390/bs16101880

**Published:** 2026-10-12

**Authors:** Jiahui Zhang, Hanxu Wang, Jiahuan Chen, Guang Zhao

**Affiliations:** 1 School of Marxism, University of Science and Technology Liaoning, Anshan 114051, China; jhzhang@ustl.edu.cn; 2 Faculty of Psychology, Tianjin Normal University, Tianjin 300387, China; psywhx@icloud.com (H.W.); cjh115660@163.com (J.C.); 3 Key Research Base of Humanities and Social Sciences of the Ministry of Education, Academy of Psychology and Behavior, Tianjin Normal University, Tianjin 300387, China; 4 Tianjin Social Science Laboratory of Students’ Mental Development and Learning, Tianjin 300387, China

**Keywords:** location probability learning, feature–spatial mapping, N2pc, LPC, visual search

## Abstract

Visual statistical learning enables humans to extract regular information from the environment, thereby optimizing the allocation of attentional resources. Previous studies have demonstrated that the visual system can learn the spatial probability of target occurrence and feature-specific spatial regularities for distractors. It remains unclear how target feature–location contingencies guide search when high-probability locations have equal overall target probability, or how such learning is reflected in ERP activity. The present study used a visual search paradigm that manipulated target-location probability and feature–spatial matching relationships, while simultaneously recording EEG data. Behavioral results showed that reaction times were significantly faster when targets appeared at feature-matched high-probability locations than when they appeared at feature-mismatched high-probability locations or low-probability locations. However, analyses by target feature showed that the feature-matching advantage was observed for color targets but not for shape targets. Electrophysiological results showed no significant differences in N2pc amplitude among the three conditions; when averaged across target features, LPC amplitudes were significantly smaller in both high-probability location conditions than in the low-probability location condition, but did not differ significantly between feature-matched and feature-mismatched high-probability locations when averaged across target features. These findings provide evidence that feature–spatial statistical regularities can influence visual search, while the feature-matching advantage was specific to color targets in the present study.

## 1. Introduction

An abundance of stimuli saturates our external environment. The selective deployment of our limited cognitive resources to focus on the stimuli that matter most is paramount to our survival. Fortunately, the world around us is not a chaotic mess but is instead highly organized. Through statistical learning, the regularities embedded in the environment can be extracted and utilized to facilitate cognitive processing (Fiser & Lengyel, 2022; Frost et al., 2019; Goujon et al., 2015). Previous studies have shown that the spatial regularity of targets can be learned, leading to improved search performance when targets appear at the high-probability target locations (HPTL) compared to the low-probability target locations (LPTL). This process is known as target location probability learning or probability cuing (Geng & Behrmann, 2002, 2005; Golan & Lamy, 2024; Jiang et al., 2013, 2014, 2018; Xu et al., 2023; Zhang & Carlisle, 2023).

Previous studies have shown that spatial probability influences target selection and distractor suppression (Failing et al., 2019b; Theeuwes et al., 2022). Feature-specific spatial learning has also been demonstrated for distractors (Failing et al., 2019a). In real-world environments, the spatial distribution of objects is often closely linked to their identities or features; for instance, a knife is more likely to be found in a kitchen, and a pedestrian is more likely to appear on a sidewalk (Brady & Oliva, 2008; Kershner & Hollingworth, 2022; Torralba et al., 2006). Pure location probability learning, which treats all targets equivalently regardless of their features, cannot fully capture this richer environmental structure. Here, we tested a target feature–location contingency at two locations with equal overall target probability. This design separates the feature-matching comparison from general spatial probability.

However, even if the behavioral effect of feature–spatial mapping learning is observed, the processing mechanism underlying this search advantage remains unclear. Regarding how implicit statistical regularities facilitate visual search, previous studies have typically interpreted the behavioral benefits of probability learning as reflecting changes in attentional priority. According to this view, regularity information establishes a proactive attentional bias, such that high-probability locations or features receive greater attentional weight during early spatial selection (Ferrante et al., 2018; Theeuwes et al., 2022; B. Wang & Theeuwes, 2018). Yet, recent studies suggest that the effects of probability learning may not arise solely from early attentional selection; statistical regularities may also influence later processing stages after attentional selection, such as target confirmation, template matching, response selection, or working-memory updating (Dolci et al., 2024; S. Wang et al., 2023; Zhou et al., 2023). From this perspective, feature–spatial mappings may facilitate search by improving the matching efficiency between the target and internal templates or learned probabilistic representations, without necessarily altering the initial spatial attentional weight (S. Wang et al., 2023).

The present study recorded electrophysiological activity while participants performed the visual search task, aiming to explore the underlying neural correlates. The present study focused on two ERP components that are respectively associated with early attentional selection and later target-related processing. Specifically, the N2pc component is a posterior contralateral negativity elicited by target stimuli and is commonly regarded as an electrophysiological marker of spatially selective attentional allocation. It can therefore be used to examine whether feature–spatial mapping learning modulates early target selection (Eimer, 1996; Luck & Hillyard, 1994). In contrast, the late positive component (LPC) has been associated with later processes such as target evaluation, stimulus significance processing, context updating, and memory-related updating. It can therefore be used to examine whether statistical learning influences target-related processing after attentional selection (Polich, 2007; Rugg & Curran, 2007; S. Wang et al., 2023). By examining both the N2pc and LPC, the present study aimed to determine whether the behavioral advantage produced by feature–spatial mapping learning arises from early spatial attentional selection, later target-related processing, or both.

To address these questions, the present study adopted a modified visual search paradigm in which both target location probability and the match relation between target features and spatial locations were manipulated. Specifically, two high-probability target locations were established, and the overall probability of target occurrence was equated across these two locations. Critically, for a given target feature, the target was more likely to appear at one high-probability location than at the other. Thus, each target feature had its own feature-matched high-probability target location, whereas the other high-probability location served as the feature-mismatched high-probability target location for that feature. This design allowed us to examine whether individuals could acquire more fine-grained target feature–spatial mapping regularities while controlling for general spatial probability. If the feature–spatial mapping regularities were learned, the search performance should be better when the target appeared at feature-matched HPTL compared to feature-mismatched HPTL. In this case, we could further determine the electrophysiological correlates of the feature-specific location probability learning by checking the difference in the amplitude of N2pc and LPC between the feature-matched high-probability location and feature-mismatched high-probability location conditions.

## 2. Materials and Methods

### 2.1. Participants 

No directly comparable effect-size estimate was available for target feature–location mapping learning. We therefore assumed Cohen’s f = 0.35 for sample-size planning, informed by related statistical-learning research (Failing et al., 2019a). A G*Power 3.1.9.7 (Heinrich Heine University Düsseldorf, Düsseldorf, Germany) analysis for the main effect of Target Location Probability on behavioral reaction times in a three-level repeated-measures ANOVA (α = 0.05, power = 0.95) yielded a target sample of 24 participants. A total of 38 participants (20 females and 18 males, aged 18–23 years) from Tianjin Normal University participated in this experiment (Approval No. 2024030805). Data from 4 participants in the original sample were excluded from formal analysis due to excessive electrophysiological data noise (more than 30% of the trials were deemed undesirable segments). The final sample comprised 34 participants. All participants had normal or corrected vision. They participated voluntarily and received financial compensation. All participants signed informed consent forms before the experiment. This study was approved by the local institution’s ethics committee.

### 2.2. Apparatus and Materials

This experiment was conducted in a noise-shielded room. Participants were approximately 80 cm from the monitor. The experimental procedure was controlled using E-Prime 3.0. Stimuli were presented on a 19-inch LED monitor (1024 × 768 pixels resolution, 75 Hz refresh rate). To minimize artifact contamination of the data, participants were instructed to keep their heads still during the task and were encouraged to relax appropriately during rest intervals. The search interface contained eight stimuli. Seven of these were gray circles (approximately 1.2 × 1.2°, RGB: 165/165/165), serving as distractions. The remaining stimulus was either a green circle (approximately 1.2 × 1.2°, RGB: 0/123/0) or a gray square (approximately 1.2 × 1.2°, RGB value 165/165/165). All stimuli were evenly distributed on an invisible circle with a radius of approximately 4.2°. Inside each stimulus was a gray stripe (approximately 0.7 × 0.2°, RGB: 165/165/165) randomly tilted 30° to the left or right. All stimuli were presented against a black background.

### 2.3. Design and Procedure

**Figure 1:**
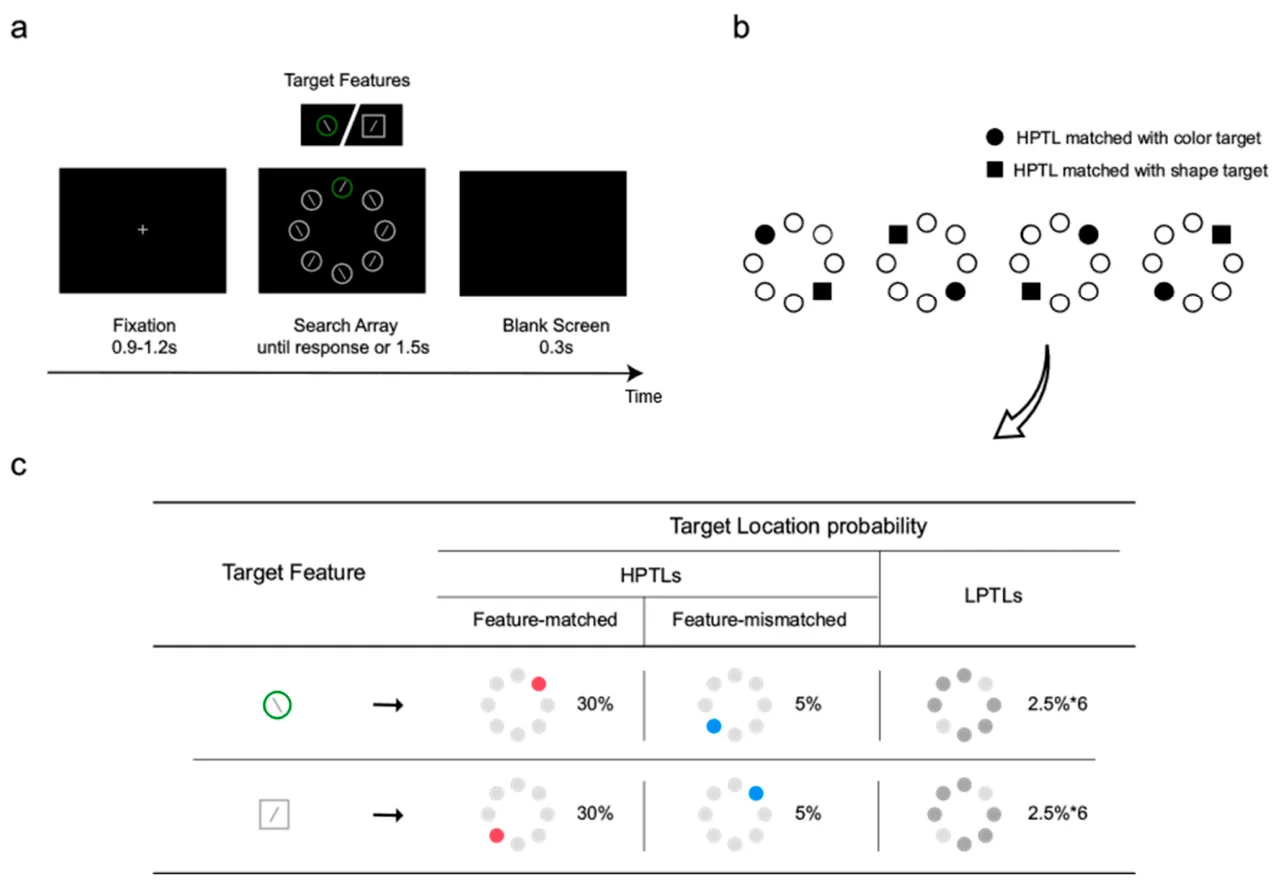
(**a**) Example of consecutive displays presented in the search task. Participants were asked to search for the unique target (either a green circle or a gray square). (**b**) Counterbalance setting. (**c**) Illustration of the feature-specific location probability. Red dots indicate feature-matched high-probability target locations (30%), blue dots indicate feature-mismatched high-probability target locations (5%), and gray dots in the LPTL column indicate the remaining six low-probability target locations (2.5% each).

As shown in Figure 1a, each trial began with the presentation of a fixation cross between 0.9 s and 1.2 s, then the search array was presented. Participants were asked to find the target and respond according to the orientation of the bar inside it by pressing the ‘j’ key with the right hand if the bar was oriented to the right or pressing the ‘f’ key with the left hand if the bar was oriented to the left. The search array was replaced by a blank screen once a response was detected or the presentation duration reached 1.5 s. Then, a blank screen was presented for 0.3 s as an inter-trial interval.

The target in our search task differed from non-targets based on either color or shape dimensions. We categorized the two kinds of targets as ‘color targets’ and ‘shape targets’. Both types of targets appeared with equal probability. Within the task, there were eight potential locations for target appearance, two out of which were high-probability target locations (HPTLs). The probability that the target appeared at each HPTL was 35%. The remaining six locations were all low-probability target locations (LPTLs). The probability that the target appeared at each LPTL was 5%. Crucially, target features were associated with different HPTLs. To be specific, the probability that a given feature appeared at one HPTL was larger (feature-matched HPTL, 30%) than the other (feature-mismatched HPTL, 5%). For example, for one participant, if the color target was matched with HPTL A, the probability of the color target appearing at A would be 30%. Correspondingly, the probability of the color target appearing at the other HPTL, B, would be 5%. Similarly, the probability of the shape target appearing at B would be 30%, and at A, it would be 5%. For the LPTLs, there was no feature-specific spatial regularity. The probability of two features appearing at each LPTL was both 2.5%. Note that the probability of a given feature appearing at a feature-mismatched HPTL was higher than at an LPTL. This alteration was made to increase the number of trials in the feature-mismatched HPTL condition to obtain a more reliable N2pc. Each search display contained only one target. Consequently, whenever the target appeared at an LPTL, both HPTLs were occupied by distractors.

The HPTLs were at maximum distance from each other (i.e., they were on the opposite sides of the imaginary circle). To analyze lateralized electrophysiological indicators, two HPTLs in our experiment were placed laterally. The HPTL pair, as well as the match relation between target feature and HPTL, was counterbalanced across participants (see Figure 1b).

To permit analysis of lateralized ERP components, the two HPTLs were placed at lateral diagonal positions. The two diagonal HPTL pairs were crossed with the two feature-to-HPTL assignments, yielding the four counterbalancing configurations in Figure 1b; participants were assigned as evenly as possible. The four EEG-based exclusions were determined solely by data quality, independent of configuration, and all four configurations remained represented in the final sample. Behavioral analyses included targets at all eight locations. ERP analyses included only the four lateral diagonal positions (two per visual field); targets at the vertical- and horizontal-meridian positions were excluded from both N2pc and LPC analyses, so that the same lateral position set was used for both components, allowing unambiguous contra-/ipsilateral classification.

All participants initially completed 32 practice trials. Four participants whose accuracy was below 90% in the first practice block completed a second block of 32 trials, for a total of 64 practice trials. Participants proceeded to the formal experiment after the experimenter confirmed that they understood the task and their practice-block accuracy reached at least 90%. There was no regularity in practice trials. The formal experiment consisted of 6 blocks of 120 trials. Participants were provided with feedback on their mean accuracy and response times (RTs) to enhance their engagement with the task. Following a short rest period, the subsequent block commenced. Participants were not informed of any existence of regularity.

### 2.4. EEG Recording and Preprocessing

The electroencephalogram (EEG) was recorded in an electrically shielded, soundproof booth from a 64-channel cap (Neuroscan system) with electrodes positioned according to the international 10–20 system. Impedance values were kept below 5 kΩ during the experiment. All channels were bandpass filtered from 0.01 to 100 Hz and recorded with a 1000 Hz sampling rate.

Offline preprocessing of the EEG data was conducted utilizing the EEGLAB Toolbox (Delorme & Makeig, 2004). After being down-sampled to 500 Hz, a high-pass filter at 0.1 Hz was applied to EEG data to remove slow signal drifts, and a low-pass filter at 30 Hz was applied to remove high-frequency activity caused by muscle and environmental noise. The continuous EEG signal was then segmented into epochs from 200 ms before search onset to 800 ms after search display onset. Each epoch was baseline-corrected relative to a pre-stimulus interval of −200 to 0 ms. Epochs severely contaminated by noise were manually inspected and removed, resulting in an average of 7.74% of trials being identified and removed due to artifact contamination. Malfunctioning electrodes marked as bad by the experimenter during recording were spherically interpolated. Then, all data was re-referenced to the average of the left and right mastoids (M1 and M2). Independent component analysis (ICA) was subsequently applied to identify and remove ocular components associated with both eye blinks and horizontal eye movements based on their characteristic frontal scalp distributions and time courses. After ICA, epochs in which the voltage at any electrode exceeded ±80 μV were rejected. After restricting ERP analyses to the four lateral diagonal positions, only trials with correct behavioral responses were included. The final dataset included an average of 402.2 artifact-free trials for the feature-matched HPTL condition, 66.8 for the feature-mismatched HPTL condition, and 65.4 for the LPTL condition. These values corresponded to retention rates of approximately 93.1%, 92.8%, and 90.8%, respectively, relative to the design-based trial counts.

## 3. Statistical Analysis

### 3.1. Behavioral Data

Trials with incorrect responses or with response times more than 2.5 standard deviations away from the participant mean reaction time were excluded for further analysis. Mean RTs were then submitted to a one-way repeated-measures ANOVA with the within-subject factor Target Location Probability (feature-matched HPTL, feature-mismatched HPTL, LPTL). RTs were additionally submitted to a 2 (Target Feature: shape vs. color) × 3 (Target Location Probability) repeated-measures ANOVA to examine whether the pattern differed between target-feature dimensions. Accuracy was analyzed only with the one-way ANOVA, as overall accuracy was near ceiling (96.7%) and did not differ across Target Location Probability conditions; the 2 × 3 breakdown was therefore not performed for accuracy. To rule out that the RT effects reflected intertrial repetition priming, the one-way RM ANOVA on RTs was repeated after excluding the following, respectively: (a) trials with target-location repetition from the immediately preceding trial, (b) trials with target-feature repetition, and (c) trials with either repetition (i.e., the union of the two exclusion criteria). Greenhouse–Geisser correction was applied when sphericity was violated, and Bonferroni-corrected pairwise comparisons were used for post hoc tests.

### 3.2. ERP Analysis

#### 3.2.1. N2pc Analysis

Following previous studies (Dolci et al., 2024; van Moorselaar & Theeuwes, 2023), the parieto-occipital electrodes (PO7 and PO8) were selected for the analysis of the target-elicited N2pc. Lateralized difference waves were computed by subtracting the ipsilateral waveforms from the corresponding contralateral waveforms. Based on visual inspection of the grand-averaged contralateral-minus-ipsilateral difference waves across all participants, the N2pc time window was defined as 220–280 ms post-search-array onset. Finally, the mean amplitude of the N2pc difference wave was extracted within this 220–280 ms window for each participant and condition.

#### 3.2.2. LPC Analysis

For the analysis of the late positive component (LPC), parietal electrodes (P3/P4, PO3/PO4, and PO7/PO8) were selected (S. Wang et al., 2023). Previous visual-search studies have examined posterior LPC activity within a broader late interval extending from approximately 450 ms onward (S. Wang et al., 2023; S. Wang & Woodman, 2024). Guided by this literature and visual inspection of the grand-average waveform collapsed across conditions, the time window for LPC analysis was set from 450 to 550 ms following the onset of the search array. The mean LPC amplitude was calculated by averaging the amplitudes across the specified electrodes and the defined time window.

## 4. Results

### 4.1. Behavioral Results

#### 4.1.1. Accuracy

**Figure 2:**
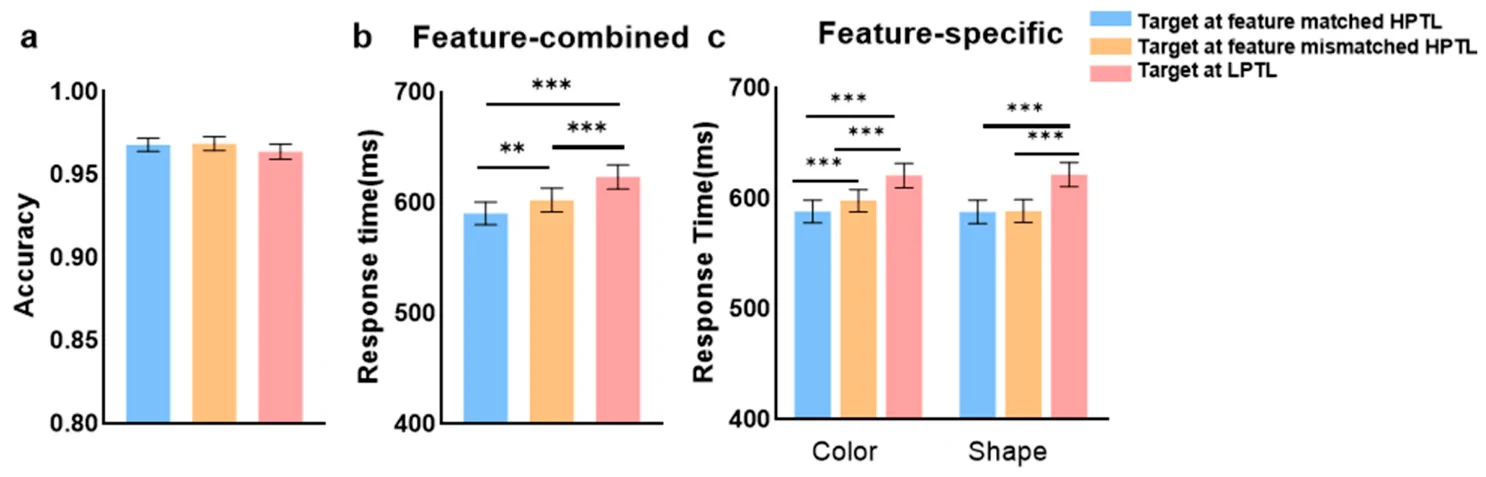
(**a**) Average accuracy. (**b**) Average response time. (**c**) The reaction time results of repeated measures ANOVA tests performed on probability location and target features respectively (2 × 3). ** *p* < 0.01; *** *p* < 0.001.

The overall mean accuracy was 96.7%. The mean accuracy for each condition is illustrated in Figure 2a. A one-way repeated-measures analysis of variance (ANOVA) with target location probability (Feature-Matched HPTL, Feature-Mismatched HPTL, LPTL) as the within-subject factor was conducted on the accuracy data. Mauchly’s test indicated that the assumption of sphericity had been violated (*p* = 0.003); therefore, the degrees of freedom were corrected using Greenhouse-Geisser estimates. The results revealed no significant main effect of target location probability, *F*(1.52, 50.16) = 1.290, *p* = 0.278, η*_p_*^2^ = 0.038. This indicates that participants’ accuracy did not differ significantly across the different probability conditions.

#### 4.1.2. Response Time

The results of RTs were shown in Figure 2b,c. The one-way RMANOVA with the within-subject factor of Target Location Probability (feature-matched HPTL, feature-mismatched HPTL, LPTL) revealed a significant main effect, *F*(2,66) = 123.99, *p* < 0.001, η*_p_*^2^ = 0.79. Post hoc tests revealed that the RTs were significantly shorter when the target appeared at the feature-matched HPTL (*M* = 594.33, *SE* = 9.62) relative to both the feature-mismatched HPTL (*M* = 606.90, *SE* = 9.95), *t*(33) = −3.37, *p* = 0.006, *d_z_* = 0.58, RTs were also significantly shorter at the feature-matched HPTL than at the LPTL (*M* = 627.90, *SE* = 10.31), *t*(33) = −13.10, *p* < 0.001, *d_z_* = 2.25. The RTs were also significantly shorter in the feature-mismatched HPTL condition than in the LPTL condition, *t*(33) = −11.11, *p* < 0.001, *d_z_* = 1.91.

To examine whether this pattern differed by the feature dimension defining the target, a 2 (Feature Dimension: shape vs. color) × 3 (Target Location Probability) RM ANOVA was conducted. The main effect of Feature Dimension was not significant, *F*(1, 33) = 0.39, *p* = 0.538, η*_p_*^2^ = 0.012, but the main effect of Target Location Probability was significant, *F*(2, 66) = 119.78, *p* < 0.001, η*_p_*^2^ = 0.784, and was qualified by a significant interaction, *F*(2, 66) = 3.47, *p* = 0.037, η*_p_*^2^ = 0.095. For color targets, RTs were shorter at feature-matched HPTLs than at feature-mismatched HPTLs, *t*(33) = −4.15, *p* = 0.001, *d*_z_ = 0.71, and LPTLs, *t*(33) = −10.21, *p* < 0.001, *d*_z_ = 1.75, and shorter at feature-mismatched HPTLs than at LPTLs, *t*(33) = −8.46, *p* < 0.001, *d*_z_ = 1.45. For shape targets, RTs did not differ between feature-matched and feature-mismatched HPTLs, *t*(33) = −0.18, *p* = 1.000, *d*_z_ = 0.03, though both were shorter than at LPTLs (both *t*s ≥ 9.07, both *p*s < 0.001). Thus, the feature-matching benefit was evident for color targets but not for shape targets.

**Figure 3:**
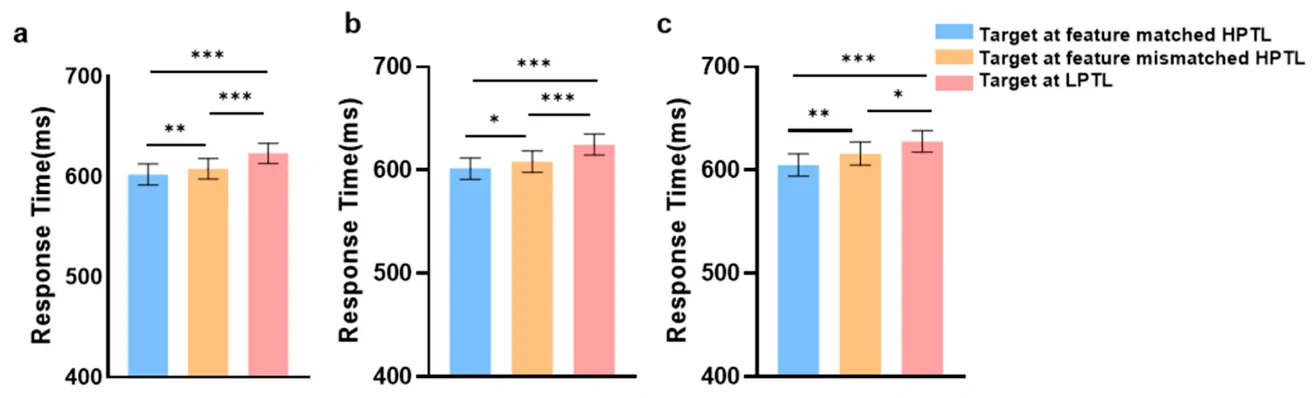
Mean response times (ms) across location conditions after controlling for intertrial repetition priming: (**a**) Location-repeat trials excluded. (**b**) Feature-repeat trials excluded. (**c**) Trials with either location-repeat or feature-repeat excluded. * *p* < 0.05; ** *p* < 0.01; *** *p* < 0.001.

To rule out that the RT advantage reflected inter-trial repetition priming, the above one-way RM ANOVA was repeated after excluding, respectively, (a) trials with target-location repetition, (b) trials with target-feature repetition, and (c) trials with either repetition, relative to the immediately preceding trial. The corresponding mean RTs are shown in Figure 3. In all three analyses, the main effect of Target Location Probability remained significant (location-repetition excluded: *F*(1.61, 53.27) = 52.24, *p* < 0.001, η*_p_*^2^ = 0.613, *M*s = 603.40/609.17/624.45 ms, *SE*s = 10.29/9.95/9.87; feature-repetition excluded: *F*(2, 66) = 37.63, *p* < 0.001, η*_p_*^2^ = 0.533, *M*s = 603.86/610.51/627.09 ms, *SE*s = 10.29/10.30/10.01; both excluded: *F*(2, 66) = 21.76, *p* < 0.001, η*_p_*^2^ = 0.397, *M*s = 606.01/616.85/628.64 ms, *SE*s = 10.43/11.00/10.03, for feature-matched HPTL/feature-mismatched HPTL/LPTL, respectively). RTs remained shortest at the feature-matched HPTL and longest at the LPTL across all three analyses, and Bonferroni-corrected pairwise comparisons remained significant in every case (all *p*s ≤ 0.021). These results indicate that the RT advantage for feature-matched HPTLs cannot be fully explained by immediate feature or location repetition priming.

### 4.2. ERP Results

#### 4.2.1. N2pc Results

**Figure 4:**
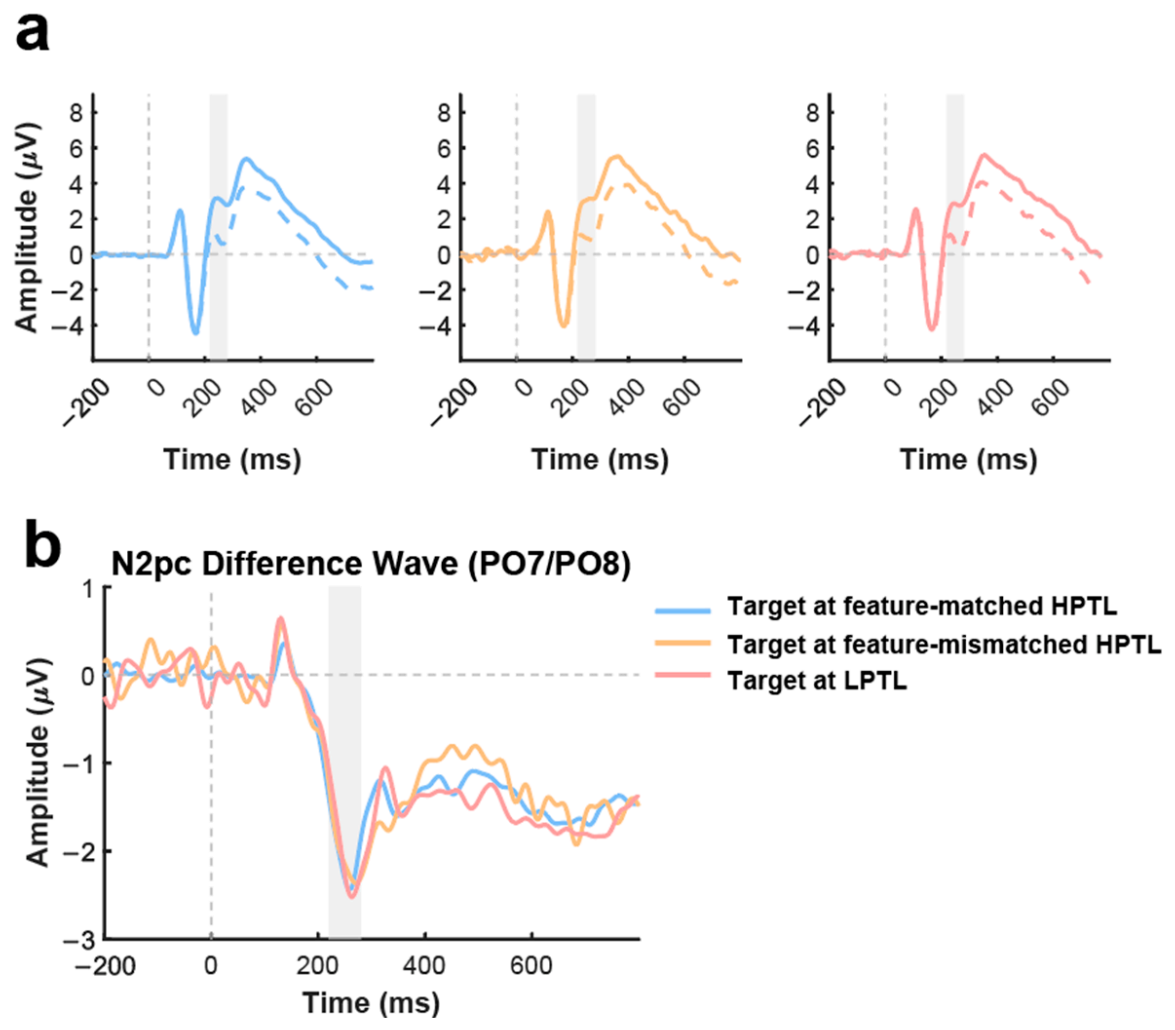
(**a**) Raw N2pc waveform (PO7/PO8 electrodes). Dashed lines represent the signal from the electrode opposite the target, and solid lines represent the signal from the electrode on the same side of the target. The gray shaded area represents the time window (220–280 ms) of N2pc under each condition. The left figure shows the feature-matched HPTL condition, the middle figure shows the feature-mismatched HPTL condition, and the right figure shows the LPTL condition. (**b**) N2pc difference waveform under each condition; vertical dashed lines represent the appearance of the search interface, and horizontal dashed lines represent an amplitude of 0.

The raw ERP waveforms at the electrodes contralateral and ipsilateral to the target (PO7/PO8) and the resulting N2pc difference waveforms for each condition are illustrated in Figure 4. To investigate the effect of statistical learning on attentional capture, we analyzed the mean amplitudes of the N2pc component (PO7/PO8) in the 220–280 ms time window. A one-way repeated measures ANOVA was conducted with Condition (Feature matched HPTL, Feature mismatched HPTL, LPTL) as the within-subject factor.

The results revealed no significant main effect of Condition on the N2pc mean amplitudes, *F*(2, 66) = 0.15, *p* = 0.862. Subsequent post hoc pairwise comparisons with Bonferroni correction confirmed that there were no significant differences in N2pc amplitudes between the feature-matched HPTL and feature-mismatched HPTL conditions, *t*(33) = −0.27, *p* = 1.000, *d*_z_ = 0.05; between the feature-matched HPTL and LPTL conditions, *t*(33) = 0.32, *p* = 1.000, *d*_z_ = 0.05; or between the feature-mismatched HPTL and LPTL conditions, *t*(33) = 0.48, *p* = 1.000, *d*_z_ = 0.08. These findings indicate that, in the selected time window, the N2pc component was not significantly modulated by the validity or feature-matching status of the spatial statistical learning cues.

**Figure 5:**
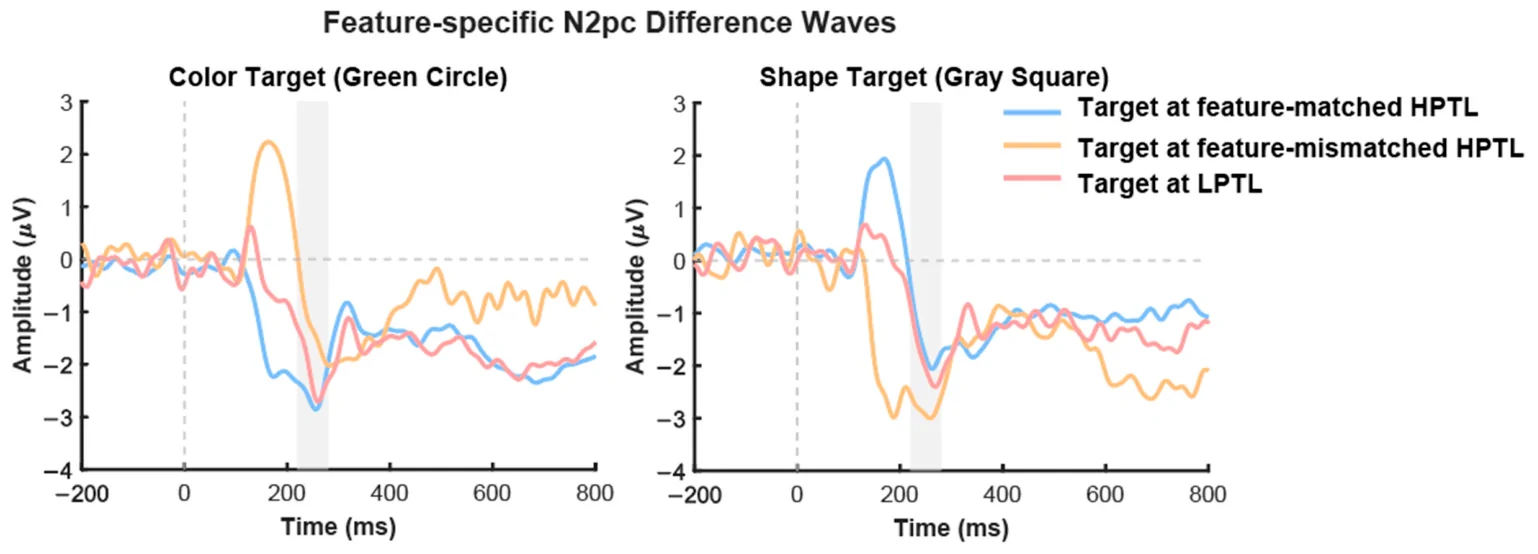
N2pc difference waves for color (**left**) and shape (**right**) targets. Shading marks the 220–280 ms analysis window. The vertical dashed line indicates search-array onset, the horizontal dashed line indicates zero amplitude, and the gray shaded area marks the N2pc analysis interval (220–280 ms). Negative values indicate greater negativity over the hemisphere contralateral to the target.

The N2pc difference waves for color and shape targets are shown in Figure 5. To examine whether the N2pc effects differed between target features, a 2 (Target Feature: shape, color) × 3 (Target Location Probability: feature-matched HPTL, feature-mismatched HPTL, LPTL) repeated-measures ANOVA was conducted. Neither the main effect of Target Feature, *F*(1, 33) = 0.007, *p* = 0.935, η*_p_*^2^ < 0.001, nor the main effect of Target Location Probability, *F*(2, 66) = 0.15, *p* = 0.862, η*_p_*^2^ = 0.004, was significant. The Target Feature × Target Location Probability interaction was also not significant, *F*(2, 66) = 1.16, *p* = 0.320, η*_p_*^2^ = 0.034. Planned analyses conducted separately for each target feature revealed no effect of Target Location Probability for color targets, *F*(2, 66) = 1.23, *p* = 0.300, η*_p_*^2^ = 0.036, or shape targets, *F*(2, 66) = 0.76, *p* = 0.472, η*_p_*^2^ = 0.023. For color targets, Bonferroni-corrected comparisons were nonsignificant for feature-matched versus feature-mismatched HPTLs, *t*(33) = −1.09, *p* = 0.846, *d*_z_ = 0.19; feature-matched HPTLs versus LPTLs, *t*(33) = −0.34, *p* = 1.000, *d*_z_ = 0.06; and feature-mismatched HPTLs versus LPTLs, *t*(33) = 1.42, *p* = 0.492, *d*_z_ = 0.24. The corresponding comparisons for shape targets were also nonsignificant, *t*(33) = 0.93, *p* = 1.000, *d*_z_ = 0.16; *t*(33) = 0.60, *p* = 1.000, *d*_z_ = 0.10; and *t*(33) = −0.95, *p* = 1.000, *d*_z_ = 0.16, respectively.

#### 4.2.2. LPC Results

**Figure 6:**
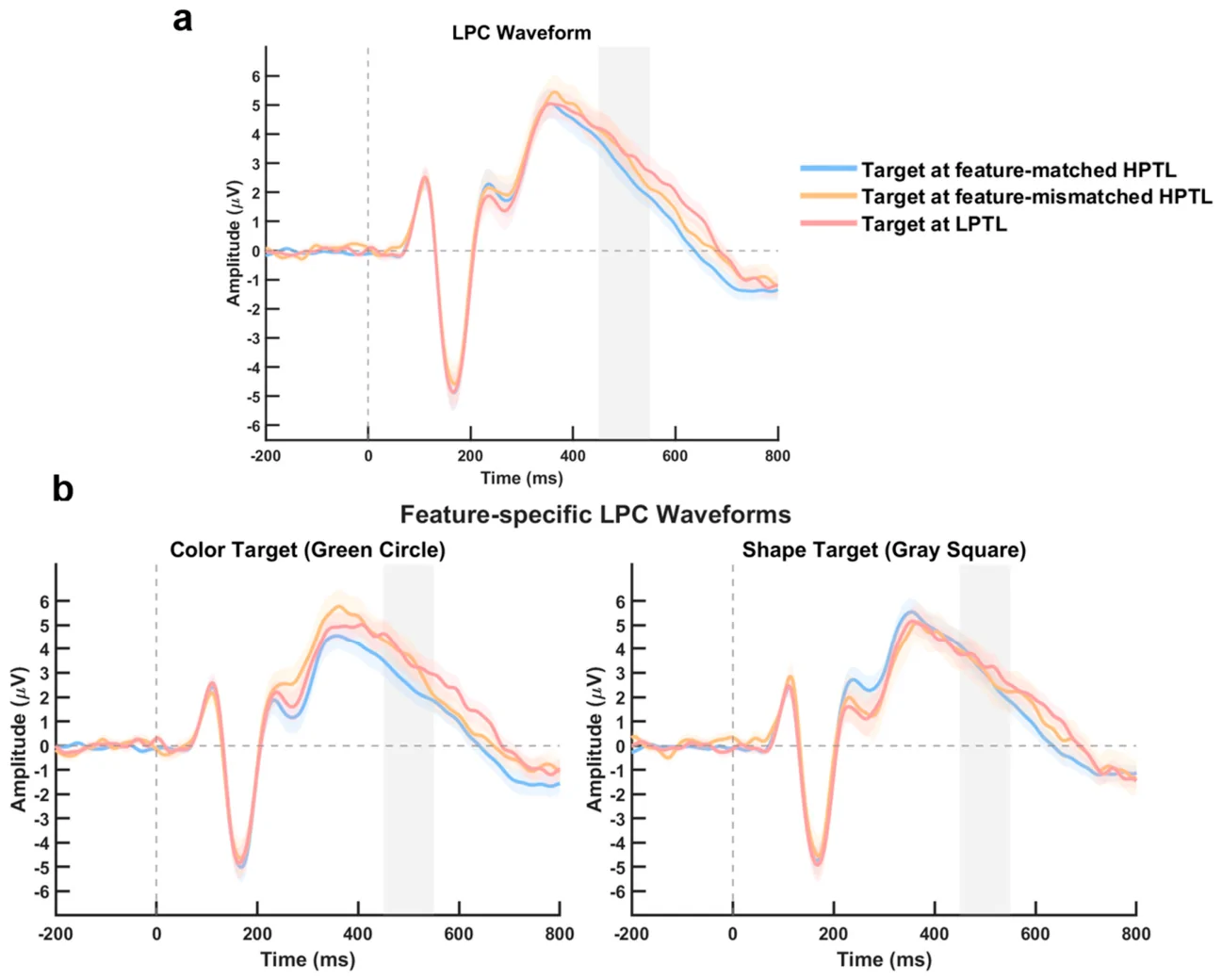
The results of LPC. (**a**) Grand-average LPC waveforms across the non-lateralized posterior ROI comprising P3/P4, PO3/PO4, and PO7/PO8 for the feature-matched HPTL, feature-mismatched HPTL, and LPTL conditions. (**b**) Feature-specific LPC waveforms, shown separately for shape targets (gray squares; right) and color targets (green circles; left). Shaded areas indicate ±1 SEM. The vertical dashed line marks search-array onset (0 ms), the horizontal dashed line indicates zero amplitude, and the gray shaded interval denotes the LPC analysis window (450–550 ms).

The results of LPC were shown in Figure 6. For the LPC, a one-way repeated-measures ANOVA with Target Location Probability (feature-matched HPTL, feature-mismatched HPTL, and LPTL) as the within-subject factor revealed a significant main effect of condition, *F*(2, 66) = 6.75, *p* = 0.002. The LPC amplitude showed a graded numerical pattern across conditions, with the smallest amplitude in the feature-matched HPTL condition (*M* = 1.78, *SE* = 0.39), a slightly larger amplitude in the feature-mismatched HPTL condition (*M* = 1.90, *SE* = 0.50), and the largest amplitude in the LPTL condition (*M* = 2.59, *SE* = 0.47). Bonferroni-corrected pairwise comparisons further showed that both the feature-matched HPTL and feature-mismatched HPTL conditions elicited significantly smaller LPC amplitudes than the LPTL condition, *t*(33) = −3.42, *p* = 0.005, *d*_z_ = 0.59, and *t*(33) = −2.86, *p* = 0.022, *d*_z_ = 0.49, respectively, whereas the two HPTL conditions did not differ from one another, *t*(33) = −0.52, *p* = 1.000, *d*_z_ = 0.09. This pattern suggests that the learned regularities may modulate later target-related processing, but the absence of a significant difference between the feature-matched and feature-mismatched HPTL conditions provides limited evidence for a clearly feature-specific late-stage effect.

A 2 (Target Feature: shape, color) × 3 (Target Location Probability: feature-matched HPTL, feature-mismatched HPTL, LPTL) repeated-measures ANOVA on LPC mean amplitudes revealed no significant main effect of Target Feature, *F*(1, 33) = 0.52, *p* = 0.477, η*_p_*^2^ = 0.015, but a significant main effect of Target Location Probability, *F*(2, 66) = 6.79, *p* = 0.002, η*_p_*^2^ = 0.171. The Target Feature × Target Location Probability interaction was not significant, *F*(2, 66) = 2.17, *p* = 0.123, η*_p_*^2^ = 0.062. Planned analyses conducted separately for each target feature revealed a significant effect of Target Location Probability for color targets, *F*(2, 66) = 11.74, *p* < 0.001, η*_p_*^2^ = 0.262, but not for shape targets, *F*(2, 66) = 0.78, *p* = 0.460, η*_p_*^2^ = 0.023. For color targets, LPC amplitudes were smaller at feature-matched than feature-mismatched HPTLs, *t*(33) = −2.70, *p* = 0.033, *d*_z_ = 0.46, and smaller at feature-matched HPTLs than at LPTLs, *t*(33) = −6.05, *p* < 0.001, *d*_z_ = 1.04; the feature-mismatched HPTL–LPTL comparison was not significant, *t*(33) = −1.60, *p* = 0.356, *d*_z_ = 0.27. For shape targets, none of the pairwise comparisons was significant after Bonferroni correction: feature-matched versus feature-mismatched HPTLs, *t*(33) = 0.45, *p* = 1.000, *d*_z_ = 0.08; feature-matched HPTLs versus LPTLs, *t*(33) = −0.80, *p* = 1.000, *d*_z_ = 0.14; and feature-mismatched HPTLs versus LPTLs, *t*(33) = −1.46, *p* = 0.461, *d*_z_ = 0.25.

## 5. Discussion

The present study aimed to examine whether individuals can acquire joint mapping regularities between target features and specific spatial locations during visual search, and further to explore whether this learning effect is expressed as a modulation of early spatial attentional selection or as a change in later target-related processing. To this end, we introduced manipulations of target-location probability and feature–spatial matching relationship in a visual search paradigm, while simultaneously recording EEG data to track its neural time course. At the behavioral level, participants showed a clear feature–spatial matching advantage: when the target appeared at a high-probability location that matched its feature, reaction times were significantly faster than when the target appeared at a feature-mismatched high-probability location or at a low-probability location. At the electrophysiological level, the results showed a pattern that was not completely consistent with the behavioral effects: N2pc amplitudes did not differ significantly among the three probability-location conditions; LPC amplitudes, by contrast, showed that both high-probability location conditions were smaller than the low-probability location condition, but there was no significant difference between the feature-matched and feature-mismatched high-probability locations. Together, these electrophysiological results suggest that the feature–spatial mapping learning effect did not show a clear modulation at the stage of early spatial attentional selection reflected by the N2pc; in contrast, the LPC results may more likely reflect the influence of general spatial probability on later target-related processing.

The behavioral results of the present study answered the question of whether joint mapping regularities between target features and specific spatial locations can be learned. Under the premise that the overall probability of target occurrence at the two high-probability locations was controlled to be equal, the reaction-time gradient across the three experimental conditions indicates that the visual system did not merely learn spatial-level location probability, but instead acquired a more fine-grained conditional probability bound to specific features. This finding extends the research framework of the classic location probability learning paradigm. In previous classic studies, for example, Geng and Behrmann (2002, 2005) and Jiang et al. (2013), researchers typically set up a hidden high-probability location in the visual search display, such that the probability of the target appearing at that location was much higher than at other locations. These studies consistently found that participants responded faster to targets appearing at the high-probability location. However, what these classic paradigms manipulated was only spatial probability; that is, regardless of the physical feature of the target, as long as it appeared at the high-frequency location, it could obtain a processing advantage. This means that traditional statistical learning, to some extent, places greater emphasis on learning spatial location itself, while less often designing conditional bindings between target features and spatial locations (Miller, 1988; Chun & Jiang, 1998; Brady & Chun, 2007). In the present study, however, the overall probability of target occurrence at the two HPTLs was 35%, composed of a crossed combination of 30% feature-matched targets and 5% feature-mismatched targets. Therefore, when participants’ reaction times in the 30% matched condition were observed to be significantly faster than those in the 5% mismatched condition, this advantage could not be explained by the simple spatial expectation that the target appeared more frequently at this location.

The feature-matching benefit was detected for color targets but not for shape targets. The color target was a green circle among gray circles, whereas the shape target was a gray square among gray circles. Color and shape can differ in how strongly they guide visual search (Theeuwes, 1992; Wolfe, 2021). In this display, color may have made the learned feature–location relation more effective during search. Shape discrimination may have placed greater demands on target identification, reducing the observed matching benefit. Neither salience nor feature-extraction demands were measured directly, so these explanations remain tentative. Moreover, matched and mismatched trials used the same physical target within each feature condition; salience alone cannot explain their reaction-time difference. The nonsignificant shape comparison does not establish that shape–location learning was absent.

Although the behavioral data indicated that feature–spatial mapping regularities can be learned, the present study did not observe significant differences in N2pc amplitude among the three target feature–spatial probability location conditions. On the one hand, the N2pc, as a contralateral-minus-ipsilateral EEG difference wave, depends strongly on the degree of spatial lateralization of the stimulus (Luck & Hillyard, 1994; Eimer, 1996). In the present experiment, the two HPTLs were placed on the left and right diagonals. Because the overall probability of target occurrence at these two locations was the same, they may have simultaneously acquired relatively high baseline spatial attentional weights in the visual system. Existing studies have shown that statistical regularities can influence visual selection by changing the spatial priority map, allowing high-probability locations to obtain higher selection weights or high-probability distractor locations to be suppressed (Ferrante et al., 2018; Theeuwes et al., 2022; Duncan et al., 2023; van Moorselaar et al., 2021). Therefore, when the target appeared at the HPTL on one side, regardless of whether its feature matched or not, the high-probability location on the opposite diagonal side might also maintain a certain degree of spatial priority. Activity at that location could reduce condition differences in the contralateral-minus-ipsilateral N2pc through bilateral cancellation, but this explanation remains untested. Each display contained one target and seven distractors. When the target appeared at an LPTL, both HPTLs contained distractors; when it appeared at one HPTL, the other contained a distractor. The available trials therefore cannot isolate the contribution of the HPTL opposite the target to the lateralized ERP. In addition, the feature-mismatched HPTL condition contained substantially fewer trials than the feature-matched HPTL condition, which may have reduced the reliability of feature-specific ERP estimates. Because HEOG was not recorded, small residual horizontal eye movements toward the target cannot be fully excluded. Future studies could vary whether a distractor appears at that location while keeping the rest of the display comparable.

On the other hand, feature-specific location probability learning could also influence processing after early spatial selection. From the perspective of neural computation, the feature–spatial mapping regularity in the present study is not simply overall spatial frequency information, but a conditional statistical structure that binds specific target features with spatial coordinates (Fiser & Aslin, 2001; van Moorselaar & Theeuwes, 2023). The behavioral advantage associated with this joint regularity could also emerge after the target feature has been extracted, potentially involving feature–location matching, internal template confirmation, or task-related decisions.

The present study also focused on the LPC component. The LPC has been associated with target evaluation, stimulus salience, and working memory retrieval (Polich, 2007; Rugg & Curran, 2007; Fields, 2023). The present study found that, regardless of whether the target feature matched its high-probability location, the LPC amplitudes elicited under both high-probability location conditions were significantly smaller than those under the low-probability location condition. This pattern suggests that spatial probability may also influence late target-related processing. It is consistent with evidence that statistical learning can affect visual search beyond early spatial selection (S. Wang et al., 2023; Dolci et al., 2024).

However, the behavioral feature-matching advantage was not accompanied by an overall LPC difference between the matched and mismatched HPTLs when color and shape targets were combined. In this compound search task, participants first located the target and then judged the orientation of the line inside it. The common LPC reduction at both HPTLs may reflect less demanding evaluation of target location (Polich, 2007). Once the defining feature is identified, a match with its learned location could help confirm the target and facilitate the subsequent orientation judgment. This offers a possible explanation for the additional RT advantage without assuming that it must produce a uniform LPC difference across target features. For color targets, the LPC was also smaller at the matched than at the mismatched HPTL. This finding may reflect use of a learned feature–location expectation during later target-related processing, consistent with the view that visual statistical learning can involve higher-order structural relations (Fiser & Aslin, 2001; Brady & Oliva, 2008; van Moorselaar & Theeuwes, 2023). However, the Target Feature × Target Location Probability interaction was not significant, so the present data do not establish a stronger LPC matching effect for color than for shape. The link between feature–location matching and target confirmation remains an interpretation of the behavioral and LPC patterns, rather than a processing stage isolated by these measures.

## 6. Conclusions

The present study demonstrates that the visual system can acquire fine-grained joint mappings between target features and spatial locations, at least for color-defined targets. Behaviorally, individuals responded significantly faster to targets whose features matched their high-probability locations. However, analyses by target feature showed that this feature-matching advantage was observed for color targets but not for shape targets. Neurally, N2pc amplitudes did not differ across conditions, suggesting that this learning effect did not modulate early spatial attentional selection. Both high-probability locations elicited smaller LPC amplitudes than low-probability locations, consistent with an effect of spatial probability on later target-related processing. 

## Data Availability

Data will be made available upon request.
